# Development of Patient Resource for Lecanemab APOE Genetic Testing

**DOI:** 10.1002/alz.083716

**Published:** 2025-01-09

**Authors:** Victoria Klee, Laynie Dratch, Jamie C Fong, Abigail A Freeman, Malia C. Rumbaugh

**Affiliations:** ^1^ The Ohio State University, Columbus, OH USA; ^2^ Frontotemporal Degeneration Center, University of Pennsylvania, Philadelphia, PA USA; ^3^ Baylor College of Medicine, Houston, TX USA; ^4^ University of Wisconsin ‐ Madison, Madison, WI USA; ^5^ Indiana University School of Medicine, Indianapolis, IN USA

## Abstract

Current joint practice guidelines (PG) on genetic counseling and testing for Alzheimer’s disease (AD), published in 2011 by the National Society of Genetic Counselors (NSGC) and American College of Medical Genetics), recommend against clinical APOE genetic testing. These recommendations were largely followed, as seen in a survey of AD Research Centers in 2019 where only 7% of centers reported disclosure of APOE to research participants.

However, because the risk of amyloid related imaging abnormalities (ARIA) associated with anti‐amyloid therapy is increased for those with one or two copies of APOE e4, the FDA now endorses APOE testing for those considering this treatment. The current PG states that APOE testing should only occur within the context of genetic counseling. Because AD is a common disease, it is not feasible for genetic counselors to see every patient with MCI or early‐stage AD considering anti‐amyloid therapy. APOE testing is complex, with implications not only for the patient, but for their family members. This has created a need for patient‐facing educational materials and revised clinical guidelines, including when to refer for genetic counseling.

The NSGC Dementia Workgroup has received approval to formally update the current PG. Meanwhile, we recognize the urgent need for patient‐facing materials. We therefore developed a factsheet on APOE testing for lecanemab to be used by any provider prescribing the therapy. It has been reviewed by stakeholder and professional groups to optimize utility. Additional multimedia resources are in development.

Although there is a spotlight on APOE given the advances in anti‐amyloid therapeutics, the genetic landscape of AD is much more complicated and evolving. The future of genetic testing for AD includes broader dementia panel testing, exome and genome sequencing, proteomic testing for APOE and polygenic risk scores. Furthermore, APOE testing will likely expand to asymptomatic individuals for risk stratification and research participation. These new directions will increase the need for patient education and additional updates to the PG, as well as appropriate triage to maximize benefits from limited genetic counseling resources.

